# In silico analysis of the wild-type and mutant-type of BRCA2 gene

**DOI:** 10.1186/s12967-024-05200-z

**Published:** 2024-05-21

**Authors:** Jingjing Li, Rui Ge, Guanming Lu, Yuanxuan Cai, Yuan Teng, Zhe Fan, Liangyan Liao, Lingjie Kong, Jinze Zhang, Tao Wei, Qian Li, Tianzhu Long, Hongyan Yu, Jie Li

**Affiliations:** 1 Department of Breast and Thyroid Surgery, Guangzhou Women and Children’s Medical Center, Guangzhou Medical University, Guangdong Provincial Clinical Research Center for Child Health, Guangzhou, 510623 P.R. China; 2 Institute of Reproductive Health and Perinatology, Guangzhou Women and Children’s Medical Center, Guangzhou Medical University, Guangdong Provincial Clinical Research Center for Child Health, Guangzhou, 510623 P.R. China; 3 Department of General Surgery, Huadong Hospital Affiliated to Fudan University, 221 West Yan’an Road Jingan District, Shanghai, 200040 P.R. China; 4 Department of Breast and Thyroid Surgery, Affiliated Hospital of Youjiang Medical University for Nationalities, Baise, Guangxi 533000 P.R. China; 5 Key Laloratory of Molecular Pathology in Tumors of Guangxi, Baise, 533000 Guangxi P.R. China; 6 Guangzhou Dublin International College of Life Sciences and Technology, South China Agricultural University, Guangzhou, 510642 P.R. China; 7 Department of Bioengineering, College of Food Science, South China Agricultural University, Guangzhou, 510642 Guangdong P.R. China; 8 Department of Clinical Biological Resource Bank, Guangzhou Women and Children’s Medical Center, Guangzhou University, Guangzhou Institute of Pediatrics, Guangdong Provincial Clinical Research Center for Child Health, Guangzhou, 510623 P.R. China

**Keywords:** TNBC, BRCA2, Compound heterozygous variants, In silico snalysis, DNA repair

## Abstract

**Background:**

The aim of this study was to conduct an in silico analysis of a novel compound heterozygous variant in breast cancer susceptibility gene 2 (BRCA2) to clarify its structure–function relationship and elucidate the molecular mechanisms underlying triple-negative breast cancer (TNBC).

**Methods:**

A tumor biopsy sample was obtained from a 42-year-old Chinese woman during surgery, and a maxBRCA™ test was conducted using the patient’s whole blood. We obtained an experimentally determined 3D structure (1mje.pdb) of the BRCA2 protein from the Protein Data Bank (PDB) as a relatively reliable reference. Subsequently, the wild-type and mutant structures were predicted using SWISS-MODEL and AlphaFold, and the accuracy of these predictions was assessed through the SAVES online server. Furthermore, we utilized a high ambiguity-driven protein–protein docking (HADDOCK) algorithm and protein–ligand interaction profiler (PLIP) to predict the pathogenicity of the mutations and elucidate pathogenic mechanisms that potentially underlies TNBC.

**Results:**

Histological examination revealed that the tumor biopsy sample exhibited classical pathological characteristics of TNBC. Furthermore, the maxBRCA™ test revealed two compound heterozygous BRCA2 gene mutations (c.7670 C > T.pA2557V and c.8356G > A.pA2786T). Through performing in silico structural analyses and constructing of 3D models of the mutants, we established that the mutant amino acids valine and threonine were located in the helical domain and oligonucleotide binding 1 (OB1), regions that interact with DSS1.

**Conclusion:**

Our analysis revealed that substituting valine and threonine in the helical domain region alters the structure and function of BRCA2 proteins. This mutation potentially affects the binding of proteins and DNA fragments and disrupts interactions between the helical domain region and OB1 with DSS1, potentially leading to the development of TNBC. Our findings suggest that the identified compound heterozygous mutation contributes to the clinical presentation of TNBC, providing new insights into the pathogenesis of TNBC and the influence of compound heterozygous mutations in BRCA2.

## Introduction

Triple-negative breast cancer (TNBC) is the most aggressive subtype of breast cancer and characterized by the absence of hormone-receptor and the amplification of human epidermal growth factor receptor 2 (HER2) [1, 2]. TNBC accounts for approximately 15–20% of all breast cancer cases and is more frequently diagnosed in young women [2]. Although TNBC exhibits high heterogeneity, common characteristics of TNBC include activation of oncogenes and alterations in distinct DNA damage responses (DDRs) [3]. Compared with normal cells, TNBC cells exhibit a heightened reliance on existing repair pathways, and the limited functioning DDR pathways are vulnerable, increasing the susceptibility of TNBC to targeted drugs that address the DDR [4, 5].

Researchers previously identified breast cancer susceptibility gene 2 (BRCA2) by performing genetic linkage analysis with families affected by early-onset breast cancer who did not carry BRCA1 mutations [6, 7]. The BRCA1/2 genes are the most significant tumor suppressor genes involved in breast cancer. Mutations in these genes are frequently associated with an increased predisposition to breast and ovarian cancers. Deleterious mutations in BRCA2 were detected in 2.7% of unselected TNBCs [8, 9].

**Fig. 1:**
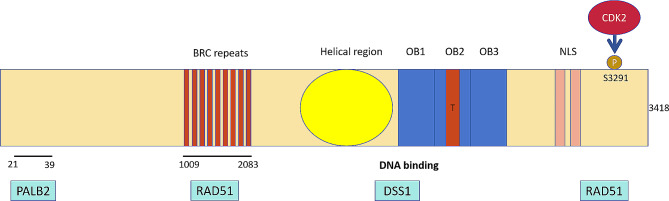
Schematic representation of the BRCA2 gene sequence and its corresponding domains [12]

The BRCA2 protein plays a key role in repairing DNA double-strand breaks (DSBs) and/or homologous recombination (HR) through interacting with RAD51 [10, 11]. Furthermore, the protein promotes recombinational DNA repair by facilitating RAD51 assembly on single-stranded DNA (ssDNA) and guiding RAD51 to ssDNA rather than to double-stranded DNA. This action enables RAD51 to displace replication protein-A (RPA) from ssDNA and stabilize RAD51-ssDNA filaments by inhibiting ATP hydrolysis (Fig. 1) [12]. Consequently, mutations in BRCA2 lead to genomic instability and the presence of genomic scars [13, 14]. Cells with wild-type BRCA2 genes repair DNA DSBs through HR; however, cells with mutations in BRCA2 or those with homologous recombination deficiency (HRD) are susceptible to poly (ADP-ribose) polymerase (PARP) inhibitors, leading to synthetic lethality [15, 16].

Numerous mutations of the BRCA2 gene have been reported and categorized into the following groups: nonpathogenic mutations and pathogenic mutations [17]. However, a common concern in genetic research is the presence of Variants of Uncertain Significance (VUS) in the BRCA2 gene [18]. These variants are genetic alterations that have been identified, but their clinical significance remains unclear. Studying the functional implications of VUS in BRCA2 patients is essential for practitioners to perform accurate risk assessments and provide counseling for individuals with hereditary breast cancers. The use of bioinformatics tools to evaluate the effects of VUS on protein stability, interactions with other molecules, or the enzymatic activity of the protein can provide valuable insights into how these mutations affect the function of the BRCA2 gene.

## Materials and methods

### Pathological analysis

Tumor tissue biopsy samples that were embedded in paraffin were evaluated and diagnosed by pathologists, and the pathological classification was confirmed in accordance with the National Comprehensive Cancer Network (NCCN) Guidelines [1].

### maxBRCA™ test

Next-generation sequencing (NGS) targeting the BRCA1/2 genes was performed using a Devyser BRCA kit (Devyser, Hägersten, Sweden) at Shuwen Guanz Diagnostic Lab Co., Ltd (Huzhou City, Zhe, China) which is a College of American Pathologists (CAP)-accredited laboratory. Sequencing reactions were conducted on the Illumina NovaSeq 6000 platform (CA, USA) using patient blood sample. The NGS data were processed using Fastp (v0.23.4) to remove low-quality data and adapters. Clean reads were mapped to the human reference genome (GRCh38/hg38) by using BWA (v0.7.17). An in-house program was used to generate run metrics including the depth of sequencing, total read count, and quality. BRCA large genomic rearrangements were also investigated. Sanger sequencing and PCR-HRMA for pathogenic or likely pathogenic variants were carried out on an ABI 3500 Genetic Analyzer (Applied Biosystems, Thermo Fisher Scientific) and a LightCycler® 480 Real Time PCR System (Roche Diagnostics, Basel, Switzerland), respectively. The results of the maxBRCA™ test were analyzed in accordance with the standards and guidelines established by the American College of Medical Genetics and Genomics/Association for Molecular Pathology (ACMG/AMP) [17].

### Sequence retrieval and the structural characteristics

The protein sequence of BRCA2 (accession number P51587) was harvested from UniProt (https://www.uniprot.org/uniprotkb/P51587) [19]. Key residues were identified using Chimera (https://www.rbvi.ucsf.edu/chimera) [20] and PyMOL (http://www.pymol.org) [21]. AlphaFold (https://github.com/deepmind/alphafold2) [22], assisted by Google’s Colab platform (https://colab.research.google.com), and homologous modeling via SWISS-MODEL (https://swissmodel.expasy.org/interactive) [23] were utilized to obtain relatively accurate structures. Subsequently, the obtained structures were evaluated using the SAVES online server (https://saves.mbi.ucla.edu), and the most highly-rated structure was selected. A single-stranded DNA fragment, obtained from Protein Data Bank (https://www.rcsb.org/), was then docked to the BRCA2 protein structure using HADDOCK [24]. Protein-ligand interactions were analyzed using the PLIP server (https://plip-tool.biotec.tu-dresden.de/plip-web/plip) [25].

## Results

### Pathological diagnosis and results of the maxBRCA™ test

**Fig. 2:**
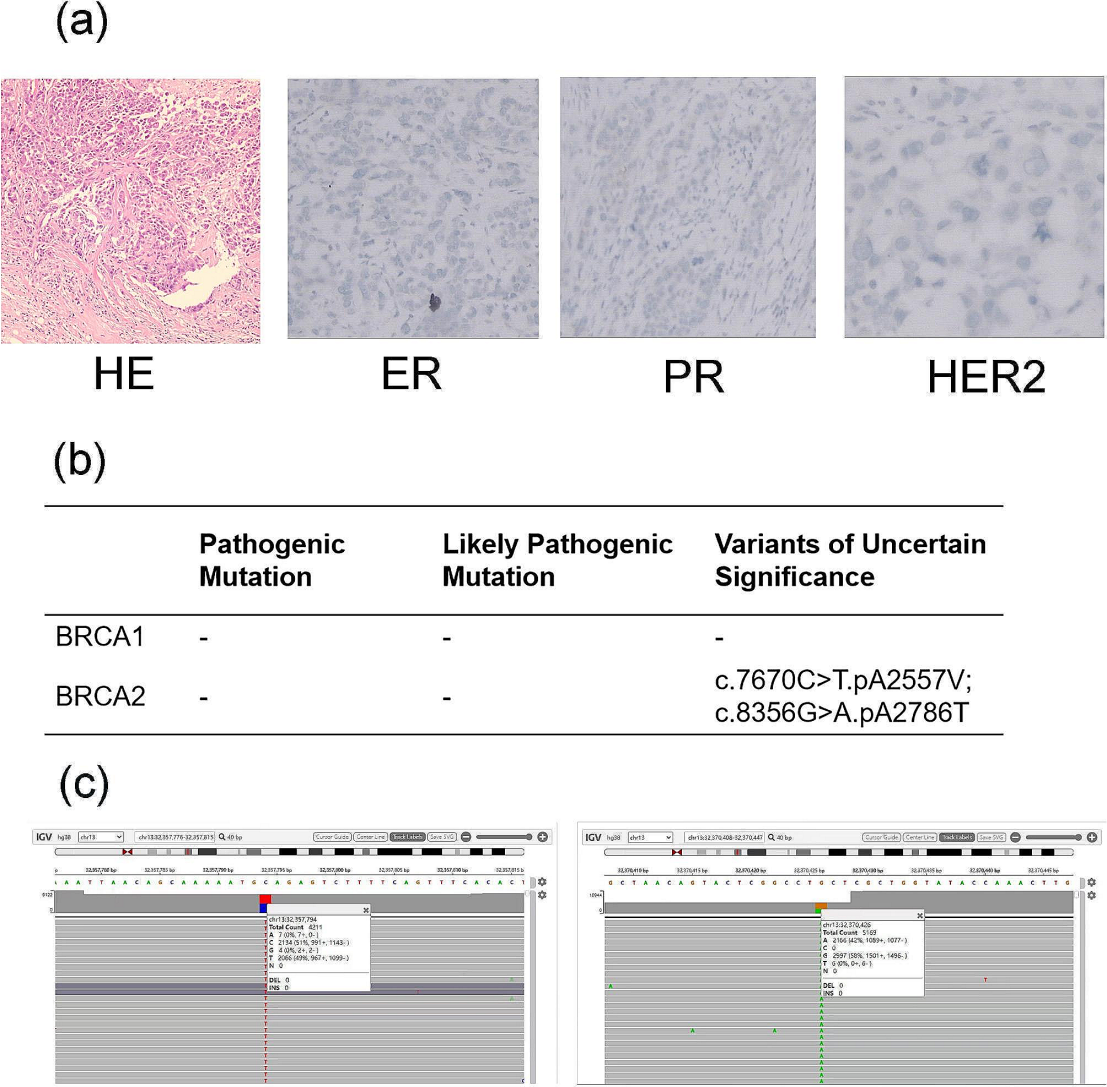
Pathological diagnosis and results of the maxBRCA™ test for the TNBC patient. (a) Images of hematoxylin-eosin staining and immunohistochemistry of estrogen receptor (ER), progesterone receptor (RP), and HER2 from the tumor sample. (b) Results of the maxBRCA™ test for the TNBC patient. (c) Variants were identified based on integrative genomics viewer (IGV).

No immunohistochemical staining revealed positive expression of ER, PR, or HER2 in the paraffin-embedded tumor tissue sample, leading to the diagnosis of triple-negative breast cancer (TNBC) in a 42-year-old Chinese woman (Fig. 2a). Furthermore, no pathogenic or likely pathogenic variants or large segment rearrangements of the BRCA1/2 genes were detected. A total of eight benign variants of the BRCA1 gene and eight benign variants of the BRCA2 gene were identified. Additionally, two variants of uncertain significance (VUS) were marked by two heterozygous mutations, denoted c.7670 C > T.pA2557V and c.8356G > A.pA2786T (Fig. 2b). As shown in Fig. 2c, the heterozygous variant c.7670 C > T (p.A2557V, Chr13: 32,357,794–32,357,794, GRch38/hg38) in the BRCA2 gene was identified with an allele frequency of 49%, while the heterozygous variant c.8356G > A (p.A2786T, Chr13: 32,370,426–32,370,426, GRch38/hg38) in the BRCA2 gene was identified with an allele frequency of 42%. These alterations had a germline derivation.

### In silico structural analysis

**Fig. 3:**
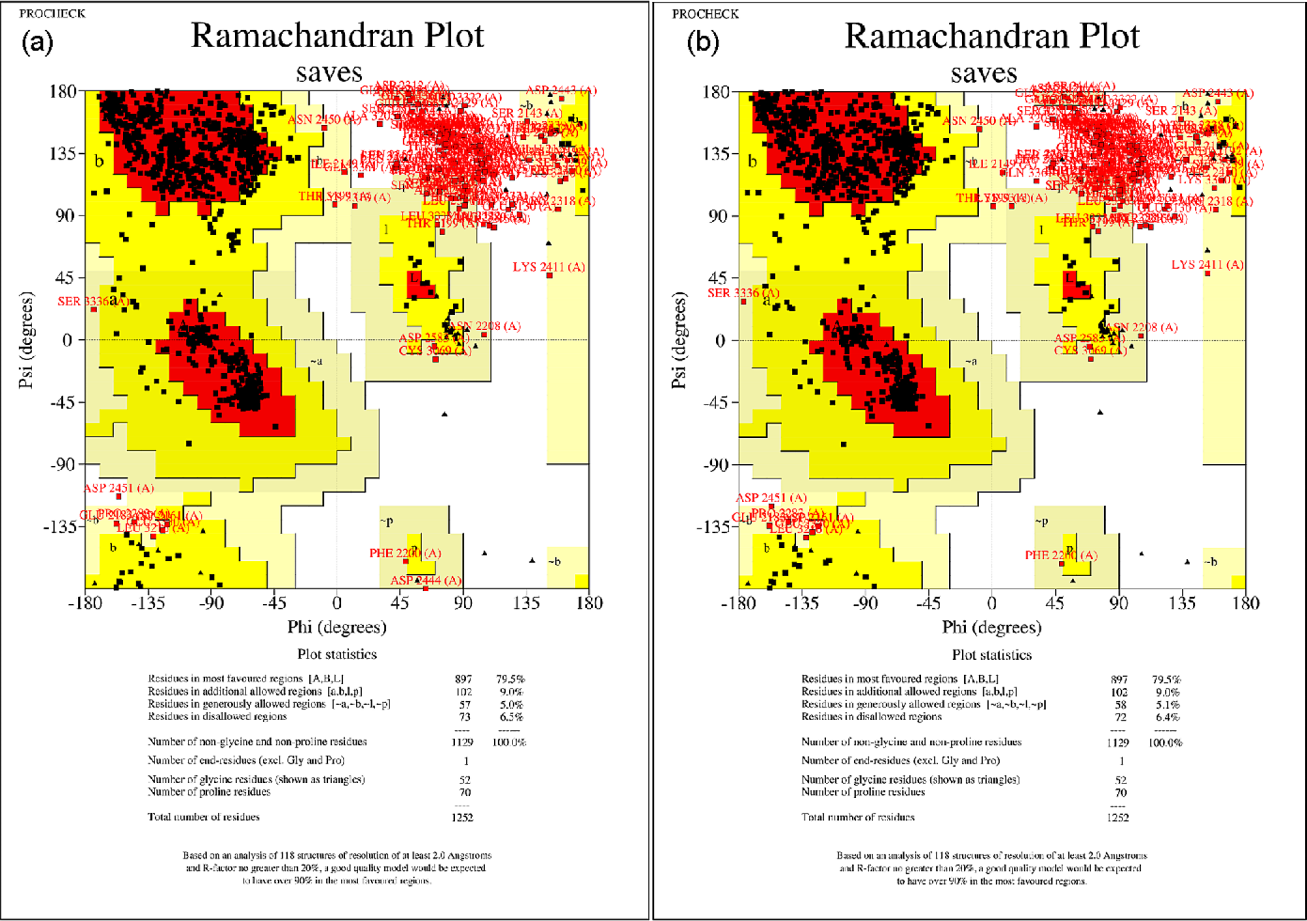
SWISS-MODEL homology modeling structure assessment. The left panel shows the wild-type BRCA2 protein structure evaluation. The right panel shows the structural evaluation of the mutant-type BRCA2 protein

To elucidate the significance of these specific mutations on the function of the BRCA2 gene, we analyzed their potential impacts on protein structure and function. Therefore, we obtained the protein sequence of BRCA2, which consists of 3418 amino acids from UniProt [19]. However, full modeling of the BRCA2 protein was not feasible due to the limitations of modeling software, which allows the entry of up to 1500 amino acids. To obtain relatively accurate structures, we used AlphaFold [22] assisted by Google’s collab arithmetic platform and conducted homologous modeling using SWISS-MODEL [23]. Vital residues were labeled using Chimera [20] and PyMOL [21]. Additionally, we selected the BRCA2 (1mje.pdb, 648 amino acids) [26] structure bound to ssDNA from the protein data bank as the reference structure, as the wild type structure did not contain a significant number of residues. The wild type and mutant structures, constructed by SWISS-MODEL and AlphaFold, were evaluated using the Ramachandran plot of PROCHECK SAVES v6.0. For further analysis, we designated the structures with the highest scores in the most favored regions as the wild type and mutant structures, respectively. Finally, we chose the structures predicted by SWISS-MODEL as our research objects due to the higher residue counts in the most favored regions (Fig. 3).

### Structure analysis

**Fig. 4:**
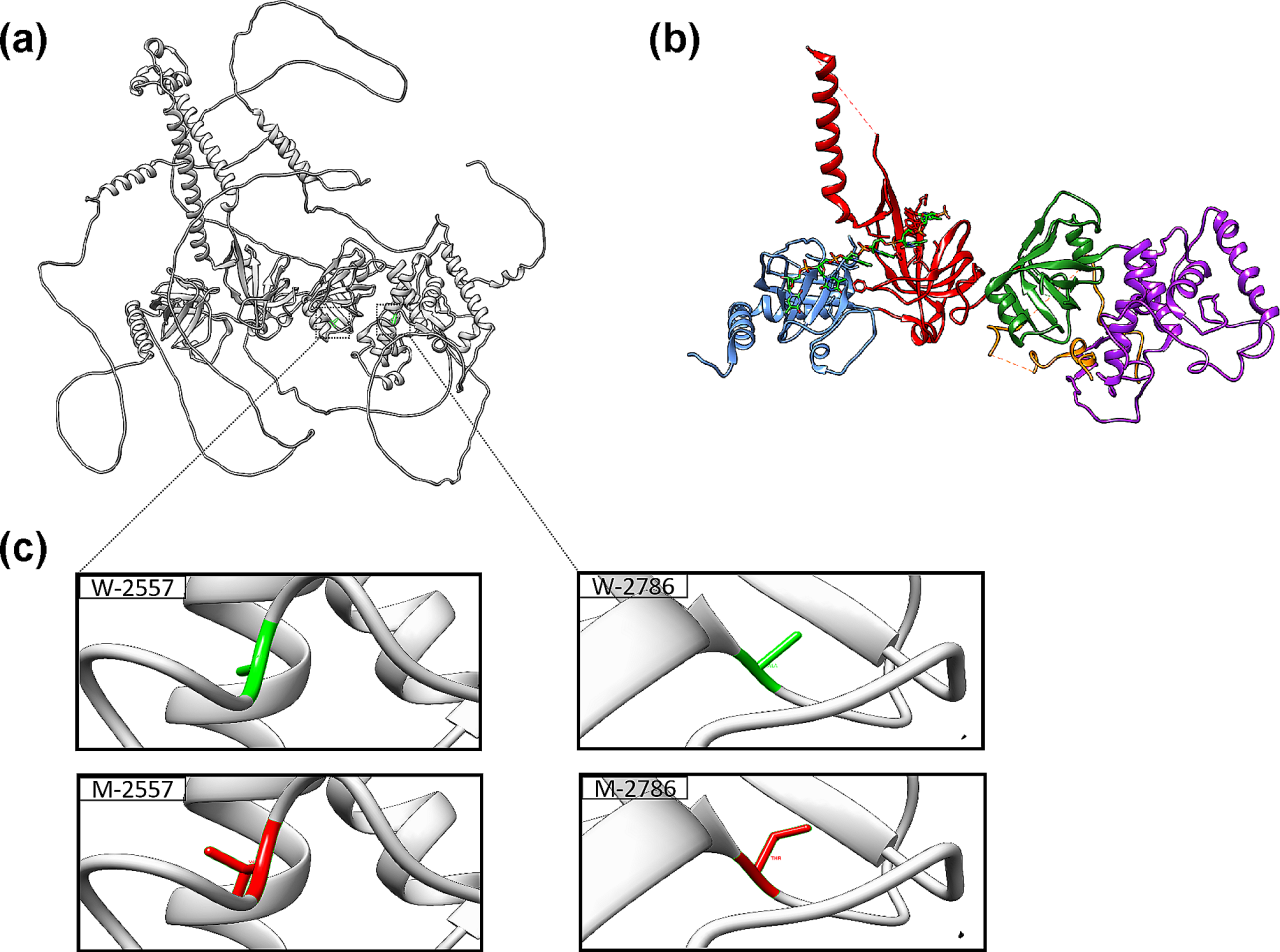
Representation of a BRCA2 predicted structure (wild type), the 1mje.pdb reference structure, and two mutation sites. (a) Structure showing that the BRCA2DBD of the predicted model is located in the middle of the whole protein, with most of the residues forming the outer long loop. Two mutant residues were located in BRCA2DBD. (b) The five subdomains that comprise the BRCA2DBD. Secondary-structure elements are colored in purple for the helical domain, forest green for OB1, red for OB2 and the tower domain, blue for OB3, green for the ssDNA fragment, and orange for DSS1. (c) Two mutation sites in the helical region and the OB1 region. Wild-type residues are labeled in green, and mutant residues are labeled in red

The SWISS-MODEL predictions indicate that the structure consists of 1252 residues, as depicted in Fig. 4a. Except for the central domain region, the overall structure primarily forms an elongated outer loop, which corresponds to the 1mje.pdb structure. Analysis of the 1mje.pdb revealed that the BRCA2 protein encompasses five domains, specifically the DNA/DSS1-binding domain (BRCA2DBD), the helical domain, oligonucleotide binding 1 (OB1), oligonucleotide binding 2 (OB2), oligonucleotide binding 3 (OB3), and a tower domain, as illustrated in Fig. 4b. The NH2-terminal segment of DSS1 traverses the helical domain and interacts with both OB1 and OB2 to form the BRCA2-DSS1 complex [12, 26]. Furthermore, the BRCA2 protein facilitates the recruitment of RAD51 filaments to ssDNA, allowing RAD51 to displace RPA from ssDNA and engage in mutual interactions during the repair process to generate a RAD51-BRCA2-DSS1 complex. This complex undergoes “error-free” homologous recombination using the sister chromatid as a template to repair damage. The wild-type protein (depicted in Fig. 4c) was compared with two amino acid mutations. First, alanine was mutated to valine at position 2557, resulting in increased hydrophilicity without observable structural changes at a smaller scale; second, alanine was mutated to threonine at position 2786, leading to enhanced hydrophobicity and a notable structural side chain alteration at a smaller scale. To further assess the impact of mutant amino acids on protein function, HADDOCK software was used to dock a ssDNA fragment bound in the 1mje.pdb structure to both the wild-type and mutant protein structures.

### BRCA2-ssDNA molecular docking and protein‒DNA interaction prediction

**Fig. 5:**
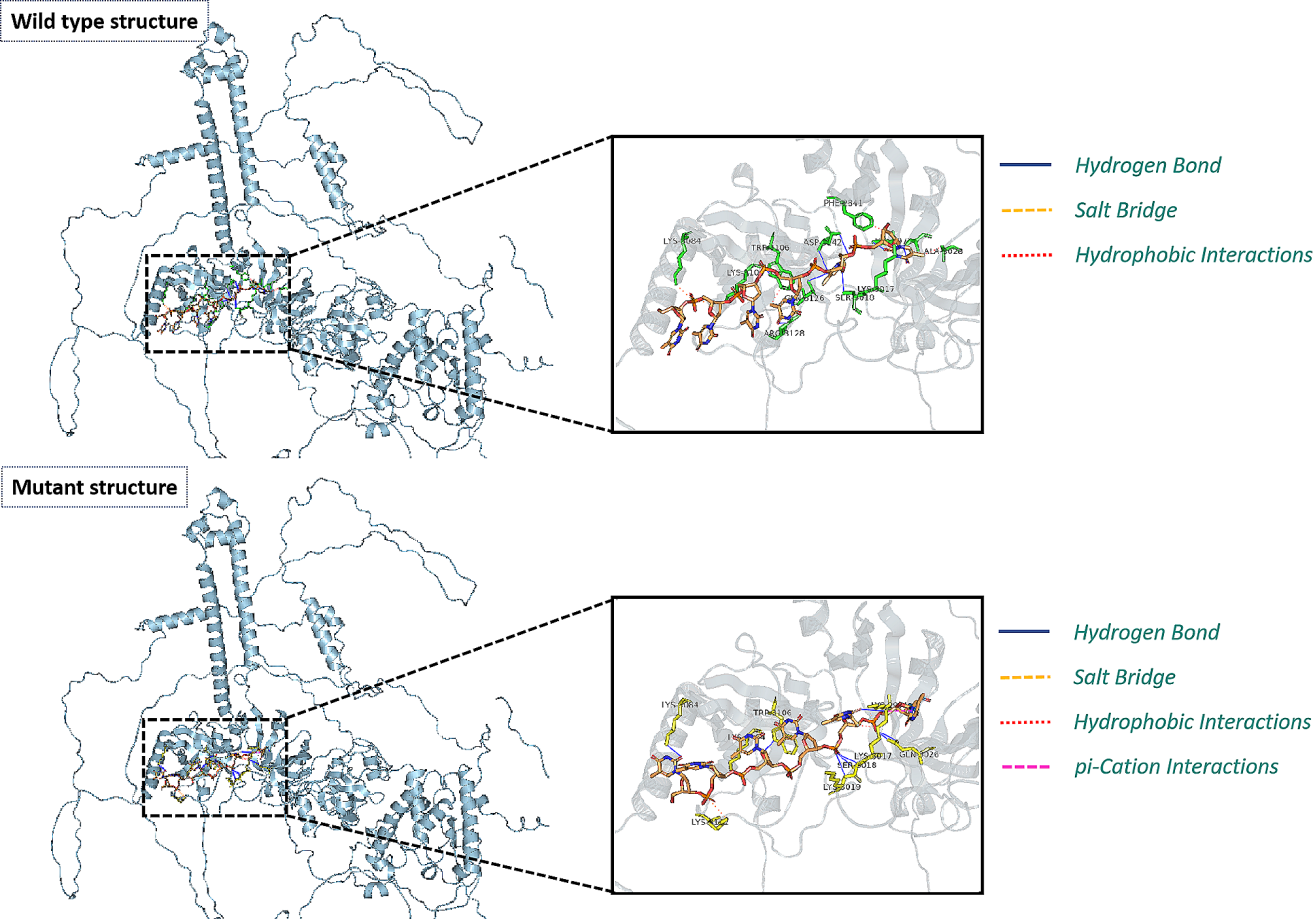
Protein–DNA interaction pattern analysis after docking by the HADDOCK algorithm. Residues of the wild-type protein structure (the picture above) that interact with DNA fragments are labeled in green, while residues of the mutant protein structure (the picture below) that interact with DNA fragments are labeled in yellow. The ssDNA fragments are labeled in orange by heteroatoms

The ssDNA fragment, which is composed of six oligomers (dT) bound in 1mje.pdb, was subjected to flexible docking with wild type and mutant structures using HADDOCK software. Amino acids within the 5 Å proximity of the protein**‒**DNA binding site were designated as active (see the official tutorial [27] for docking procedure details). As a result, two distinct conformations of the BRCA2-ssDNA complex were derived. The PLIP tool can be used to analyze protein**‒**ligand interactions, as well as interactions with DNA/RNA [25]. The prediction of protein**‒**DNA interactions in the two complex structures is illustrated in Fig. 5. The analysis of the PLIP results (presented in Table 1) revealed three modes of interaction (hydrophobic interactions, hydrogen bonds, and salt bridges) for the wild type, and four modes (hydrophobic interactions, hydrogen bonds, salt bridges, and pi-cation interactions) for the mutant BRCA2 protein with ssDNA fragments. Notably, the interacting residues in the wild- type and mutant protein structures exhibited different numbers and classes across various interaction modes.

**Table 1:** PLIP results of wild-type structure

Hydrophobic Interactions							
**Index**	**Residue**	**AA**	**Distance**	**Ligand Atom**	**Protein Atom**		
1	2841 A	PHE	3.49	12,481	7057		
2	3028 A	ALA	3.18	12,477	8990		
3	3106 A	TRP	3.29	12,413	9753		
Hydrogen Bonds							
**Index**	**Residue**	**AA**	**Distance H-A**	**Distance D-A**	**Donor Angle**	**Donor Atom**	**Acceptor Atom**
1	2971 A	LYS	3.10	3.95	139.11	8396 [N3+]	12,471 [O2]
2	2988 A	SER	2.19	2.93	132.96	8571 [O3]	12,475 [O2]
3	3017 A	LYS	2.47	3.40	149.31	8878 [N3+]	12,464 [O3]
4	3018 A	SER	2.08	2.98	153.80	8888 [O3]	12,432 [O3]
5	3106 A	TRP	2.77	3.11	100.49	9749 [Nar]	12,393 [O2]
6	3126 A	GLN	3.36	3.76	106.10	9939 [Nam]	12,443 [O2]
7	3128 A	ARG	2.49	3.03	111.65	9969 [Ng+]	12,411 [O2]
8	3142 A	ASP	2.95	3.93	175.31	10,087 [Nam]	12,443 [O2]
9	3142 A	ASP	2.87	3.68	145.50	10,093 [O3]	12,439 [O2]
Salt Bridges							
**Index**	**Residue**	**AA**	**Distance**	**Ligand Group**	**Ligand Atoms**		
1	3084 A	LYS	3.53	Phosphate	12,327, 12,327, 12,328, 12,329, 12,330, 12,326		
2	3104 A	LYS	4.82	Phosphate	12,327, 12,327, 12,328, 12,329, 12,330, 12,326		
3	3104 A	LYS	3.33	Phosphate	12,359, 12,359, 12,358, 12,360, 12,361, 12,362		

As shown in Table 1, the wild-type protein exhibited three hydrophobic interactions with DNA bases, involving residues 2841PHE, 3028ALA, and 3106TRP, while the mutant protein only exhibited one interaction with the bases through residue 3017LYS. Additionally, in the wild-type protein, eight residues (2971LYS, 2988SER, 3017LYS, 3018SER, 3106TRP, 3126GLN, 3128ARG, and 3142ASP) formed a total of nine hydrogen bonds with the base fragment, among which 3142ASP contributed two hydrogen bonds. In comparison, the mutant protein contained seven residues (2988SER, 3017LYS, 3018SER, 3019LYS, 3026GLN, 3084LYS, and 3106TRP), which formed nine hydrogen bonds with the bases; among these residues, 3017LYS and 3026GLN each contributed two hydrogen bonds with the base fragment (Table 2). Furthermore, the ssDNA fragment formed three salt bridges with two residues (3084LYS and 3104LYS) in the wild-type protein, in contrast to the three salt bridges formed by ssDNA with three residues (3971LYS, 3104LYS, and 3132LYS) in the mutant protein. Additionally, one pi-cation interaction was detected between the base fragment and residue 3017LYS of the mutant protein.

**Table 2:** PLIP results of the mutant-type structure

Hydrophobic Interactions							
**Index**	**Residue**	**AA**	**Distance**	**Ligand Atom**	**Protein Atom**		
1	3017 A	LYS	3.96	12,454	8881		
Hydrogen Bonds							
**Index**	**Residue**	**AA**	**Distance H-A**	**Distance D-A**	**Donor Angle**	**Donor Atom**	**Acceptor Atom**
1	2988 A	SER	2.09	3.05	170.60	12,491 [O3]	8576 [O3]
2	3017 A	LYS	2.42	3.37	152.05	8883 [N3+]	12,444 [O2]
3	3017 A	LYS	2.71	3.61	154.02	12,430 [O3]	8888 [O2]
4	3018 A	SER	1.86	2.78	157.67	8893 [O3]	12,429 [O2]
5	3019 A	LYS	2.55	3.47	154.63	8897 [Nam]	12,430 [O3]
6	3026 A	GLN	1.95	2.89	158.98	8978 [Nam]	12,462 [O3]
7	3026 A	GLN	2.43	3.33	153.67	12,462 [O3]	8977 [O2]
8	3084 A	LYS	2.83	3.67	138.37	9544 [N3+]	12,316 [O2]
9	3106 A	TRP	2.05	3.03	170.40	9754 [Nar]	12,373 [O3]
π-Cation Interactions							
**Index**	**Residue**	**AA**	**Distance**	**Offset**	**Ligand Group**	**Ligand Atoms**	
1	3017 A	LYS	3.76	1.51	Aromatic	12,472, 12,473, 12,475, 12,477, 12,479, 12,481	
Salt Bridges							
**Index**	**Residue**	**AA**	**Distance**	**Ligand Group**	**Ligand Atoms**		
1	2971 A	LYS	3.61	Phosphate	12,460, 12,460, 12,459, 12,461, 12,462, 12,463		
2	3104 A	LYS	4.09	Phosphate	12,364, 12,364, 12,363, 12,365, 12,366, 12,367		
3	3132 A	LYS	4.34	Phosphate	12,332, 12,332, 12,331, 12,333, 12,334, 12,335		

Overall, our PLIP analysis revealed that wild type and mutant proteins exhibited different interaction patterns with ssDNA fragments in terms of the number and type of interacting residues. Previous studies have demonstrated that the quantity of buried hydrophobic surfaces upon protein**‒**ligand binding serves as an optimal structural parameter associated with binding affinity, a phenomenon observed across a broad range of protein**‒**ligand complexes. Furthermore, hydrophobic interactions are a primary consideration in drug design [28]. Consequently, a decrease in the number of hydrophobic interactions strongly indicates that mutations in amino acids diminish the protein’s ability to bind ligands.

Intriguingly, the mutant protein, which is derived from homology modeling, exhibited nearly identical residue positions in the protein binding sites. It is commonly believed that mutating amino acid sites away from the protein binding site has a limited impact on the protein binding ligand. However, the five structural domains of the BRCA2DBD play a crucial role in the binding of BRCA2 to DNA and DSS1. In the HADDOCK algorithm, biochemical and biophysical interaction data, such as chemical shift perturbation data from NMR titration experiments or mutagenesis data, are employed to introduce ambiguous interaction restraints (AIRs) and drive the docking process. Unlike numerous other docking programs, HADDOCK enables molecules to undergo conformational changes during complex formation, affecting not only the side chains but also the backbone. It is possible that HADDOCK yields different docking results based on variations in sequences and structural spaces between wild-type and mutant proteins.

## Discussion

Two compound heterozygous variants of the BRCA2 gene (c.7670 C > T.pA2557V; c.8356G > A.pA2786T) were identified in TNBC patient based on the maxBRCA™ test. However, the role of VUS remains unclear. Additional biophysical analyses should be conducted to further determine and accurately predict the effects of these gene mutations.

Due to the limitations in current technology, the construction of large-scale protein structures using various modeling software involves several challenges. Additionally, due to the scarcity of financial and human resources, performing experiments at the experimental level is impractical. As a result, computational biology analysis has become a viable alternative for our research efforts.

BRCA2 protein structures consisting of 1252 amino acid residues were generated using the AlphaFold and SWISS-MODEL methods. These structures were evaluated through the SAVES server to identify the most favorable regions for further investigation. The ssDNA fragments, which were bound to the crystal structure, were then aligned to both the wild-type and mutant protein structures using the HADDOCK algorithm. Subsequently, the interaction patterns between the protein and DNA were analyzed using the PLIP tool.

The ssDNA fragment binds within the OB2-OB3 channel of the BRCA2DBD region. The mutation site at position 2557 is located within the helical region, while position 2786 is located found within the OB1 region. Surprisingly, the mutation of these amino acids does not seem to impact the binding of ssDNA within the BRCA2DBD region. Nonetheless, some studies suggest that all five structural domains of BRCA2 are crucial for its tumor suppressor function [26, 29]. These domains correspond to regions with structural roles, regions bound to DNA or DSS1, or regions on the tower’s surface. Furthermore, researchers have demonstrated that DSS1 is necessary for the stability of BRCA2 [30]. The mutations A2557V and A2786T may impact the binding between BRCA2 and DSS1, resulting in loss of biological function of the BRCA2 protein. Additionally, the protein‒DNA interaction prediction from the HADDOCK docking results indicated that these amino acid mutations might influence protein‒DNA interactions. Recently, in silico analysis provided strong evidence that A2557V and A2786T mutations are pathogenic. However, further biochemical experiments should be performed to explore the association between genotype and phenotype.

The aunt of the TNBC patient was diagnosed with breast cancer without a known BRCA phenotype. Due to germline derivation, the maxBRCA™ test should be performed with her other immediate family members. Promising results have been obtained with PARP inhibitor olaparib in the treatment of metastatic breast cancer patients with germline BRCA mutations [15, 31]. Our approach might reveal a new type of BRCA2 VUS that may benefit from olaparib treatment.

## Conclusion

By employing in silico analysis, we have successfully elucidated the impact of VUS on the function of BRCA2 protein function in a three-dimensional structure. The computational analysis of both wild types and variants revealed the deleterious nature of these mutations. The findings from this study significantly contribute to our knowledge on VUS in the BRCA2 gene. Moreover, through analyzing of the impact and domain function both prior to and following the amino acid mutation, we can clarify how the substitution of amino acids contributes to the occurrence of TNBC.

## Data Availability

The data sets generated during and/or analyzed during the current study are available from the corresponding author on reasonable request.

## Abbreviations

BRCA2: breast cancer susceptibility gene 2
TNBC: triple-negative breast cancer
PDB: Protein Data Bank
PLIP: protein-ligand interaction profiler
OB1: oligonucleotide binding 1
HER2: human epidermal growth factor receptor 2
DDR: DNA damage response
DSBs: DNA double-strand breaks
HR: homologous recombination
ssDNA: single-stranded DNA
RPA: replication protein-A
HRD: homologous recombination deficiency
PARP: poly (ADP-ribose) polymerase
VUS: Variants of Uncertain Significance
NCCN: National Comprehensive Cancer Network
NGS: Next-generation sequencing
ACMG/AMP: American College of Medical Genetics and Genomics/Association for Molecular Pathology
OB2: oligonucleotide binding 2
OB3: oligonucleotide binding 3
AIRs: ambiguous interaction restraints
IGV: integrative genomics viewer
